# Optimization of PLGA Nanoparticle Formulation via Microfluidic and Batch Nanoprecipitation Techniques

**DOI:** 10.3390/mi16090972

**Published:** 2025-08-24

**Authors:** Gül Kozalak, Salar Heyat Davoudian, Evangelos Natsaridis, Nubia Gogniat, Ali Koşar, Oya Tagit

**Affiliations:** 1 Group of Biointerfaces, Institute of Chemistry and Bioanalytics, University of Applied Sciences and Arts Northwestern Switzerland, Hofackerstrasse 30, 4132 Muttenz, Switzerland; 2 Faculty of Engineering and Natural Science, Sabancı University, Istanbul 34956, Turkey; salar.hdavoudian@sabanciuniv.edu (S.H.D.);; 3 Center of Excellence for Functional Surfaces and Interfaces for Nano Diagnostics (EFSUN), Sabancı University, Istanbul 34956, Turkey; 4 Sabanci University Nanotechnology Research and Application Centre (SUNUM), Istanbul 34956, Turkey

**Keywords:** PLGA nanoparticles, nanoprecipitation, design of experiments, microfluidics, computational fluid dynamics

## Abstract

Polymeric nanoparticles based on poly(lactic-co-glycolic acid) (PLGA) are widely used in drug delivery, yet scalable and reproducible production methods remain a major challenge. In this study, we combine experimental nanoprecipitation and computational fluid dynamics (CFD) modeling to optimize PLGA nanoparticle formulation using both traditional batch and microfluidic methods. While Design of Experiments (DoE) was used to optimize the batch process, microfluidic mixing was systematically explored by varying flow parameters such as the flow rate ratio (FRR) and total flow rate (TFR). We compared two microfluidic mixer designs with Y-junction and three-inlet junction geometries to evaluate their impact on the mixing efficiency and nanoparticle formation. Experimental results revealed that the three-inlet design produced smaller, more uniform nanoparticles with superior post-lyophilization stability. CFD simulations confirmed these findings by displaying velocity fields and PLGA concentration gradients, demonstrating significantly more homogeneous mixing and efficient interfacial contact in the three-inlet configuration. Furthermore, simulated outlet concentrations were used to predict the nanoparticle size via theoretical modeling, which closely agreed with the experimental data. This integrated approach highlights the importance of microfluidic geometry in controlling nanoparticle nucleation dynamics and provides a framework for rational design of scalable nanomedicine production systems.

## 1. Introduction

Polymeric nanoparticles (NPs) have been increasingly recognized as transformative tools in drug delivery. Their ability to enhance drug bioavailability, protect labile compounds from degradation, and minimize systemic toxicity through targeted delivery has positioned them at the forefront of personalized medicine [1]. Despite substantial preclinical success, only a limited number of nanomedicine formulations have received regulatory approval for clinical use [2]. Achieving precise and reproducible control over nanoparticle size remains a central challenge in nanoformulation development [3], emphasizing the ongoing need for robust, scalable, and reproducible manufacturing strategies to facilitate their clinical translation.

Polymeric NPs based on poly(lactic-co-glycolic acid) (PLGA) particularly stand out due to the biocompatibility, tunable degradation times, and regulatory approval of PLGA for clinical use [3,4,5]. PLGA nanoparticles are suitable for encapsulating diverse therapeutic agents, including small molecules, nucleic acids, and proteins [6], and can be administered via multiple routes such as intramuscular, pulmonary, or oral delivery [7]. Because the physicochemical properties of PLGA nanoparticles such as particle size and size distribution strongly influence their pharmacokinetics, cellular uptake, and therapeutic efficacy [8], achieving consistent control over these critical quality attributes remains a key challenge, particularly when translating laboratory-scale methods to a scalable production [9].

PLGA NPs can be prepared in a variety of ways, including emulsion, nanoprecipitation, spray drying, and solvent diffusion [10]. Among these techniques, nanoprecipitation has been commonly used due to its simplicity and efficiency particularly for encapsulating hydrophobic cargo. Nanoprecipitation relies on the rapid diffusion of a water-miscible organic solvent containing the dissolved polymer into an aqueous phase, leading to polymer precipitation and nanoparticle formation [11]. Yet, traditional batch nanoprecipitation methods are often limited by poor mixing control and variability in particle formation dynamics, resulting in broader size distributions and reduced reproducibility [12,13]. This is especially problematic during scale-up, where solvent diffusion and mixing heterogeneities lead to batch-to-batch inconsistencies and a broad particle size distribution [14,15]. To address these limitations, systematic experimental designs, such as Design of Experiments (DoE), are increasingly used to optimize process variables [13,16]. DoE provides a structured approach for identifying interactions between formulation parameters (e.g., PLGA concentration, organic-to-aqueous ratio, surfactant concentration), enabling better control over critical quality attributes [17].

In parallel, the advent of microfluidics has introduced a promising alternative for the controlled formation of nanoparticles [18,19]. Microfluidics offers unparalleled data quality in less time, and is portable, parallelizable, and digitizable, enabling complex laboratory procedures to be combined in a single instrument [20,21]. Microfluidic systems enable nanoparticle formulation with rapid and uniform mixing, precise control over flow conditions, efficient use of materials, and scalable production when operated continuously or in parallel using multiple microchannels [22,23,24]. The controlled laminar and chaotic flow environments in microfluidics promote rapid supersaturation and nucleation, often resulting in smaller and more monodisperse particles than conventional batch processes [19]. These platforms facilitate fine control over nanoparticle size and morphology by tuning variables such as flow rate ratio (FRR), total flow rate (TFR), channel geometry, and temperature [25]. Notably, staggered herringbone micromixer geometries promote chaotic advection, enhancing interfacial diffusion and improving nanoparticle uniformity [26,27].

In this study, we present two complementary strategies for PLGA nanoparticle formulation via nanoprecipitation: a traditional batch method guided by Design of Experiments (DoE) and a microfluidic system designed for precise control over mixing parameters. We also included a systematic comparison of two microfluidic mixer geometries, Y-junction and three-inlet junction, to investigate the role of inlet design on the mixing efficiency and nanoparticle formation. To deepen our understanding of the underlying flow dynamics, computational fluid dynamics (CFD) simulations were made to visualize velocity fields and concentration profiles within each mixer. By integrating experimental optimization with numerical modeling, we aim to provide a robust framework for selecting and designing nanoparticle manufacturing platforms depending on the resource availability, throughput requirements, and formulation goals, and thus propose standardized conditions for the reproducible production of PLGA-based nanoparticles suitable for drug delivery applications.

## 2. Materials and Methods

### 2.1. Materials

PLGA (Resomer RG 502 H), Mw 7000–17,000 Da, was obtained from Evonik GmbH (Darmstadt, Germany). Acetonitrile (ACN, 99.95%) was purchased from VWR International (Radnor, PA, USA). Polyvinyl alcohol (PVA, 9000–10,000 Mw, 80%, hydrolyzed) was obtained from Thermo Fisher Scientific (Waltham, MA, USA).

### 2.2. Batch Preparation of PLGA Nanoparticles with DoE

For the batch preparation of PLGA NPs, an organic phase comprising PLGA dissolved in ACN was added every three seconds dropwise to the aqueous phase containing PVA surfactant while continuously stirring at 660 rpm with a magnetic stirrer bar (3 mm × 10 mm) in a glass vial with a 17 mm outer diameter. After the complete evaporation of the organic solvent following overnight incubation, the NPs were washed twice by centrifugation at 15,000 rpm for 20 min and lyophilized using a Kühner Alpha 2-4 LD freeze-drier (Martin Christ Gefriertrocknungsanlagen GmbH, Osterode am Harz, Germany).

The design of experiments (DoE) tool Stavex was utilized to design the experiments with a focus on controlling the NP size and polydispersity index (PDI). The target values for the NP size and PDI were 200 nm and 0.1, respectively, selected based on commonly accepted criteria for enhanced circulation time, stability, and cellular uptake in drug delivery applications [19]. The dissolved amount of PLGA in the organic phase (mg), volume of the organic solvent ACN (μL), amount of PVA in the aqueous phase (*w*/*v* %), and the total volume of the aqueous phase (mL) were selected as response variables in ranges suitable for small-scale batch processes. For the organic phase, the parameters varied between 5 mg and 15 mg for PLGA, and between 100 μL and 500 μL for ACN. The set limits for aqueous phase were 1.5% to 2.5% (*w*/*v* %) PVA solution in a volume range of 1 mL to 3 mL. The initial design iteration generated 8 runs, setting the stage for the preparation of PVA solutions and polymer solutions in a systematic manner. A total of three iterations were run.

#### 2.2.1. Hydrodynamic and Statistical Analysis

To characterize the mixing regime and interpret particle formation trends, we performed dimensionless number analysis and regression modeling using data from three experimental iterations of batch nanoprecipitation.

##### Hydrodynamic Characterization

Mixing during nanoprecipitation was conducted in 5 mL cylindrical glass vials (outer diameter ≈ 17 mm) using a 10 mm × 3 mm PTFE-coated magnetic stir bar at 660 rpm. Based on this setup, the Reynolds number (*Re*) was calculated for a stirred vessel (*Re_i_*) [28] as:

(1) $${Re}_{i}=\frac{N_{i}D_{i}^{2}\rho }{\mu }$$

where ρ is the fluid density at 25 °C (997 kg/m^3^), μ is dynamic viscosity (0.89 × 10^−3^ Pa·s), *D_i_* is the impeller diameter (0.01 m), and *N_i_* is the rotational speed of the impeller (11 rev/s, corresponding to 69.12 rad/s).

The Damköhler number (*Da*), which is defined as the ratio of the characteristic mixing time (*t_mix_*) to the reaction time (*t_react_*), was calculated to evaluate the interplay between the nanoprecipitation kinetics and bulk mixing dynamics. In this context, *t_mix_* was approximated as 10/*N_i_*, which corresponds to ca. 0.91 s. The characteristic reaction time *t_react_*, which is determined by the solvent displacement and polymer precipitation, was estimated to be 0.1 s, consistent with typical timescales reported for the nanoprecipitation processes.

##### Regression Modelling of Formulation Variables

Each of three iterations consisted of eight batch formulations prepared using varying ratios of PLGA, PVA, and solvent volumes. For each run, the following derived variables were computed:

PLGA/ACN (mg/μL): PLGA concentration in organic phase
% PVA: *w/v* % of PVA in aqueous solution
Aq/Org (*v*/*v*): volume ratio of aqueous phase to organic phase

Multiple linear regression was performed independently for each iteration using PLGA/ACN ratio, %PVA, and aqueous/organic volume ratio as predictors of particle size and polydispersity index (PDI). Models were fitted using ordinary least squares (OLS) in Python (statsmodels v0.14.0). *p* < 0.05 indicated the significance of the tested parameters on the resulting NP size and PDI.

### 2.3. Preparation of PLGA Nanoparticles Using Microfluidic Mixing

A commercial microfluidic platform (Tamara, Inside Therapeutics) supplied with microfluidic chips featuring herringbone and baffle mixers were used for the preparation and optimization of PLGA NPs. In this study, the herringbone configuration was selected due to its ability to generate chaotic advection and micro-vortices within the channel, significantly enhancing mixing efficiency. This configuration incorporates a three-inlet junction, in which the organic phase flows through the central channel and is flanked by two aqueous streams, allowing for focused and rapid mixing. In parallel, a custom-built microfluidic system utilizing two syringe pumps connected to a commercial mixing chip (Fluidic 1460, Microfluidic ChipShop, Jena, Germany) was tested to prepare PLGA NPs. While the custom-made setup also utilized a microfluidic chip with herringbone configuration, the mixing of organic and aqueous phases took place through a Y-junction. The microchannel dimensions in both setups were very similar: 300 µm × 600 µm, with a herringbone groove depth of approximately 100 µm.

For the preparation of PLGA NPs using microfluidics, a 1% PVA aqueous solution was filtered through a 0.45 µm membrane. The organic phase was prepared by dissolving 10 mg of PLGA in 1 mL of acetonitrile (ACN). For the optimization of NP size and PDI, different aqueous-to-organic flow rate ratios (FRRs) and total flow rates (TFRs) were tested. While the FRRs were varied between 2:1–6:1, the tested TFR range was 2.5–15 mL/min. Following mixing, the product streams were collected in individual glass vials containing magnetic stir bars and placed on a magnetic stirrer at 300 rpm overnight to evaporate residual ACN. The following day, the nanoparticle suspensions were transferred to Eppendorf tubes and centrifuged at 15,000 rpm. The resulting supernatants were discarded, and the pellets were redispersed in 1 mL of Milli-Q water (Merck Millipore, Darmstadt, Germany) and sonicated in a bath sonicator to ensure complete dispersion. This washing step was repeated three times. Finally, the NPs were reconstituted in 1 mL of Milli-Q water and lyophilized at −80 °C for 24 h using a Kühner Alpha 2-4 LD freeze-dryer (Martin Christ Gefriertrocknungsanlagen GmbH, Osterode am Harz, Germany). 

### 2.4. Colloidal Characterization of PLGA Nanoparticles

#### 2.4.1. Dynamic Light Scattering (DLS)

The hydrodynamic diameter and polydispersity index (PDI) of the nanoparticles were determined using dynamic light scattering (DLS) on a Malvern Zetasizer ZS Nano instrument (Malvern Instruments Ltd., Malvern, UK). Measurements were performed at a NP concentration of 0.01 mg/mL, with samples prepared in MQ water and placed in disposable cuvettes. The mean and standard deviation of three measurements were used to report the NP size distribution and PDI.

#### 2.4.2. Atomic Force Microscopy

The morphological characterization of NPs was made using a multi-mode NanoScope atomic force microscope (AFM) (Bruker, Billerica, MA, USA). For sample preparation, 100 μL of 1 mg/mL particle suspension was dried on clean glass substrates, and particles were imaged in the peak-force tapping mode. Silicon nitride cantilevers with nominal spring constants of 0.4 N/m (Bruker, Billerica, MA, USA) were used for imaging at 1 Hz scan rate. 256 lines with 256 points per line were recorded during image acquisition. AFM images were analyzed using the NanoScope analysis software v3.0 (Bruker, Billerica, MA, USA).

**Figure 1:**
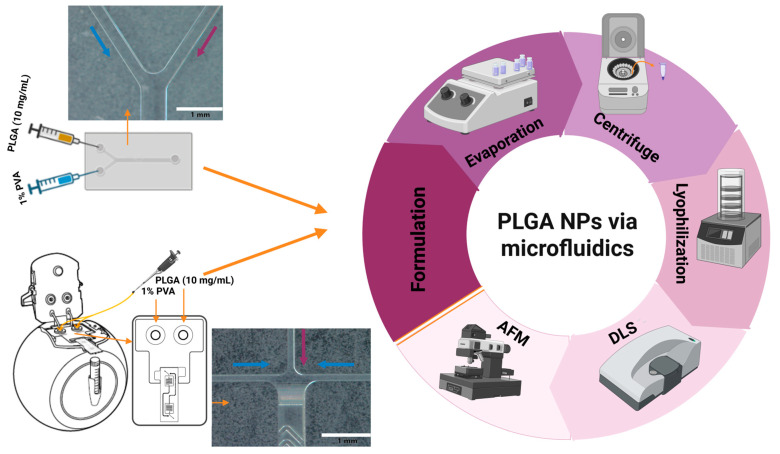
Inlet geometries of the microfluidic chips (scalebar: 1 mm) and the schematic representation of the workflow for the microfluidic preparation and characterization of PLGA NPs.

The entire schematic for the microfluidics-based preparation and characterization of PLGA NPs is illustrated in Figure 1.

### 2.5. Computational Fluid Dynamics (CFD) Analyses

**Figure 2:**
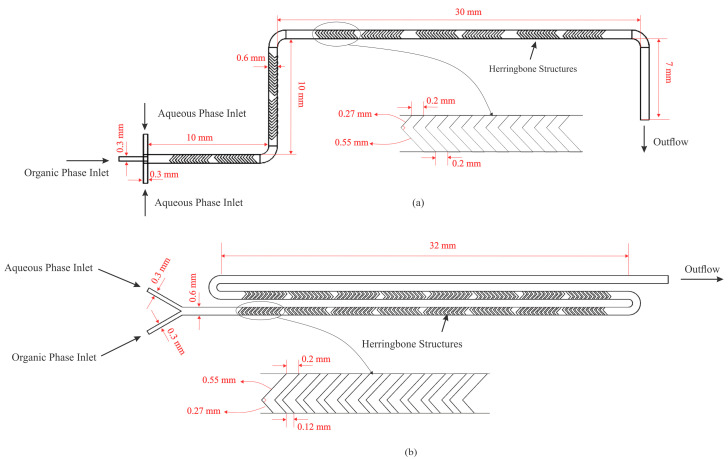
Simulation domain for (**a**) three-inlet junction, and (**b**) Y-junction mixers.

CFD simulations were performed to predict the mean diameter of PLGA nanoparticles produced by both the three-inlet-junction and Y-junction mixing geometries. Figure 2 displays schematic representations of the two systems, including the herringbone mixer geometry employed in the microfluidic device.

The fluid motion in both mixers is governed by the full Navier–Stokes equations coupled with mass conservation [29];

(2) $$\rho \frac{Du}{Dt}=-\nabla p+\nabla \cdot \left(2\mu S\right)$$

(3) $$\frac{D\rho }{Dt}+\rho \nabla \cdot u=0$$

where the material derivative $\frac{D}{Dt}=\frac{\partial }{\partial t}+u\cdot \nabla$ follows a fluid parcel as it moves through space and time. Here, ρ, **u**, p, and μ are the local fluid density, velocity vector, hydrodynamic pressure, and dynamic viscosity, respectively. S is the rate-of-strain tensor, defined as $S=\frac{1}{2}\left(\nabla u+{\left(\nabla u\right)}^{T}\right)-\frac{1}{3}\left(\nabla \cdot u\right)I$ which extracts the deviatoric (shear) part of the velocity gradients.

To predict PLGA concentration fields (and ultimately nanoparticle size), the flow field u computed [from Equations (2) and (3)] is coupled to a Transport of Diluted Species module;

(4) $$\frac{\partial c}{\partial t}+\nabla \cdot \left(-D\nabla c+uc\right)=0$$

where c is the local PLGA concentration and D its molecular diffusivity in the solvent [30].

The laminar flow and species transport boundary conditions were specified as follows. At both the organic and aqueous inlets, a velocity inlet was imposed corresponding to 0.625 mL/min and 1.875 mL/min flow rates, respectively. The outlet boundary was set to 0 Pa gauge pressure, and all channel walls were treated as no-slip. For the Transport of Diluted Species interface, the organic inlet was held at a constant PLGA concentration of 0.83 mol/m^3^, while the aqueous inlet concentration was set to zero. A convective-flux (outflow) condition was applied at the outlet to allow the PLGA concentration to exit freely, and all walls were specified as no-flux. In this study, an unstructured tetrahedral mesh with localized boundary layer refinement (three layers, first layer thickness 1 µm, growth rate 1.2) was used.

### 2.6. Statistical Analysis

Statistical analyses were performed using the GraphPad Prism 5.1 program. Unpaired t-test was used to compare two groups, and ANOVA followed by Dunnett multiple comparison tests were used to compare more than two groups to determine the significance of the difference in the mean particle size (*p* < 0.01).

## 3. Results and Discussion

### 3.1. Batch Preparation of PLGA NPs Using DoE

Batch nanoprecipitation remains one of the most accessible and widely used methods for the development of polymeric NPs due to its simplicity, low cost, and minimal equipment requirements [31]. The process relies on the rapid supersaturation of a polymer upon mixing a solvent with a non-solvent, leading to nanoparticle formation. Several formulation and process parameters, such as the polymer and surfactant concentrations, aqueous-to-organic phase volume ratio, and stirring rate critically influence the physicochemical properties of the resulting NPs [32]. However, despite its practical advantages, batch nanoprecipitation often suffers from limited control over micromixing dynamics, which can result in inconsistent particle sizes and broad polydispersity. To overcome these challenges and enhance batch process robustness, we implemented a Design of Experiments (DoE) strategy to systematically investigate the influence of key formulation variables and identify optimal conditions for producing PLGA NPs with a target size of approximately 200 nm and a polydispersity index (PDI) below 0.1. The outcomes of three sequential DoE iterations, each comprising eight formulations (*FX1*–*FX8*, where *X* corresponds to the Iteration number), are presented in Table 1. The influence of formulation parameters, such as the PLGA concentration, PVA concentration, and mixed volumes of organic and aqueous phases on NP size and PDI are demonstrated.

The first iteration explored a broad range of PLGA concentrations (5–15 mg), PVA concentrations (1.5–2.5%), and volume ratios. Particle sizes ranged from 132 nm (*F1.5*) to 281 nm (*F1.6*). A lower PLGA concentration (5 mg) combined with higher PVA (2.5%) generally resulted in smaller particles *(F1.1*, *F1.5*), while higher PLGA (15 mg) and lower PVA (1.5%) concentrations tended to increase the particle size (*F1.4*). Notably, *F1.8* produced highly polydisperse NPs as reflected by a very high PDI likely due to suboptimal micromixing at that scale. Overall, PDI values were moderately variable, with some formulations (*F1.5*, *F1.1*) yielding more monodisperse particles, and others (*F1.4*, *F1.6*) exhibiting broader distributions.

The second iteration round aimed to refine the formulation space based on promising conditions obtained from Iteration 1. Particle sizes spanned a wide range, but most formulations now yielded sizes between 134 nm (*F2.7*) and 270 nm (*F2.1*), suggesting increased consistency. The highest size and PDI (*F2.1*) were again associated with a high PLGA concentration (15 mg) in a small ACN volume (100 µL), emphasizing the influence of polymer concentration on nucleation kinetics. Importantly, most PDI values were below 0.1, indicating improved monodispersity. Interestingly, formulations *F2.3*, *F2.4*, and *F2.5* resulted in PLGA nanoparticles with comparable sizes (~194–200 nm) and low PDI values (<0.1) despite the variations in input conditions. These results suggest that increasing both the volume of aqueous phase and PVA concentration can offset the impact of higher PLGA loading, maintaining narrow particle size distributions. This highlights a balance among the polymer content, solvent ratio, and stabilizer concentration as key drivers for the nanoparticle uniformity.

In the final iteration, the PLGA concentration range was slightly narrowed (5–12 mg), while holding other parameters similar to previous iterations. The results highlighted the sensitivity of the system to organic loading, with *F3.1* and *F3.2* (100 µL ACN with 12 mg PLGA) yielding dramatically larger particles (1483 nm and 4793 nm, respectively) and very high PDIs (~0.9), indicating poor micromixing and unstable particle formation. In contrast, *F3.3–F3.6* (500 µL ACN, 12 or 5 mg PLGA) resulted in particle sizes below 210 nm with low PDIs (<0.07 for *F3.5* and *F3.6*), demonstrating that better organic dispersion and solvent mixing for this volume favored nanoprecipitation. These results affirm the critical role of the PLGA/ACN concentration and volume ratio in driving both particle size and homogeneity.

The general trends for all three iterations suggest that higher PLGA concentrations (>10 mg per 100 µL ACN) led to excessive particle growth and poor uniformity, consistent with fast supersaturation and inadequate stabilization. On the other hand, higher PVA concentrations (2.5%) often yielded lower PDI values, reflecting better surface stabilization during nucleation. A comparison of formulation compositions shows that *F1.1*, *F2.8*, and *F3.6* from iterations 1, 2, and 3, respectively, were prepared under identical conditions. These three independent batches yielded a mean hydrodynamic diameter of 144 ± 11 nm (SD), corresponding to a coefficient of variation (CV) of 7.6%, which is consistent with the expected variability of manual dropwise batch mixing.

To better understand the role of formulation parameters and mixing dynamics on nanoparticle characteristics in the batch method, we conducted hydrodynamic and statistical analyses of the results obtained in three DoE iterations. Based on the dimensions of stir bar (10 mm × 3 mm), and a stirring speed of 660 rpm, we first estimated the Reynolds number (*Re*) for a stirred vessel (*Re_i_*) to be approximately 7743, indicating a transition to near-turbulent regime, which is quite favorable for rapid micromixing in nanoprecipitation. Next, we calculated the Damköhler number (*Da*) assuming a mixing time of ~0.91 s and reaction time of ~0.1 s. The estimated *Da* value of 9.1 suggested that particle formation kinetics outpaces bulk mixing, supporting the hypothesis that micromixing (rather than macroscopic stirring) plays a dominant role in determining the particle nucleation and size distribution.

The subsequent regression analysis reveals that the PLGA/ACN ratio (mg/µL) was consistently the most influential predictor of both particle size (Table 2) and PDI (Table 3) across all three iterations of the batch formulations. In Iteration 1, PLGA/ACN showed a statistically significant effect on the particle size (P = 0.007), though its impact on PDI was not significant (Table 3). In Iteration 2, the PLGA/ACN ratio significantly affected both the particle size (*p* = 0.020) and PDI (*p* = 0.009), while it remained a strong determinant of both metrics (*p* = 0.004 for size, *p* = 0.001 for PDI) (Table 2 and Table 3) in Iteration 3. In contrast, the PVA concentration (%PVA) and aqueous-to-organic volume ratio (Aq/Org) generally showed no significant effect except for Iteration 3, where a higher aqueous-to-organic ratio significantly correlated with smaller particle sizes (*p* = 0.039) (Table 2). The model fit was strong for the particle size in all iterations (R^2^ = 0.84–0.91), while fit for PDI varied, being weak in Iteration 1 (R^2^ = 0.17) and much stronger in Iterations 2 and 3 (R^2^ = 0.90–0.97) (Table 2 and Table 3).

These results highlight the dominant role of the polymer concentration at the phase boundary in controlling the nanoprecipitation outcomes, suggesting that local supersaturation dynamics, rather than the bulk stabilizer content, is the primary influencer in particle size and uniformity in batch-stirred systems.

**Figure 3:**
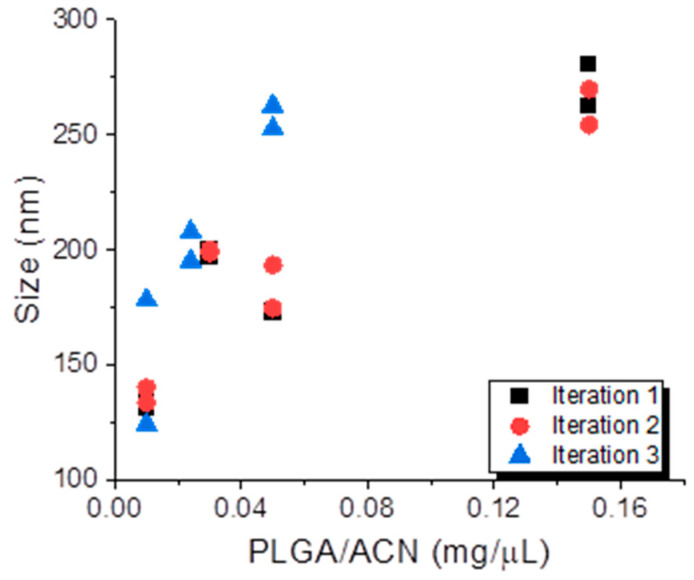
The effect of the PLGA/ACN ratio on the nanoparticle size across three DoE iterations. Nanoparticle size (nm) is plotted as a function of PLGA concentration in acetonitrile (mg/μL) for Iteration 1 (black squares), Iteration 2 (red circles), and Iteration 3 (blue triangles).

Across the three DoE iterations, a progressive improvement in the model performance was observed, particularly for particle size prediction. This trend reflects refinement in the experimental design; as such, Iteration 1 likely explored a broader and more variable parameter space, capturing both optimal and suboptimal conditions. In contrast, Iterations 2 and 3 appear to have focused on a narrower, more effective range of formulation parameters. As a result, the predictive power of the models improved, especially in Iteration 3, where both the particle size and PDI were strongly correlated with the PLGA/ACN ratio (Figure 3).

Overall, the DoE method allows for efficient evaluation of multiple variables, including the PLGA concentration, PVA content, and organic-to-aqueous phase ratio, while minimizing the number of experimental runs. This strategy not only provides statistical insight into the relationships between formulation parameters and NP properties but also enables the development of an optimized batch protocol that approaches the precision typically achieved with microfluidic systems.

### 3.2. Microfluidic Preparation of PLGA NPs

The microfluidic-based preparation of PLGA nanoparticles via nanoprecipitation is based on the same principles as the batch method [33,34], but offers inherently more efficient mixing. In particular, staggered herringbone structures in microchannels enhance mixing even under laminar flow conditions by inducing chaotic advection and transverse flow patterns, which promote faster and more uniform reactant interaction and result in controlled nucleation, narrower particle size distribution, and high monodispersity in the nanoparticles [35,36,37,38]. Furthermore, microfluidic systems offer reduced reaction times, increased yields, and scalability, making them well-suited for large-scale production [27]. In this study, we utilized a commercial microfluidic device equipped with a mixing chip featuring a three-inlet junction to systematically investigate the effects of the flow rate ratio (FRR) and total flow rate (TFR) on the size and PDI of PLGA nanoparticles.

The compositions of the organic and aqueous phases were fixed at 10 mg/mL of PLGA in ACN and 1% PVA, respectively, while the FRR and TFR were systematically varied. Initially, the TFR was held constant at 5 mL/min, and FRRs of 2:1, 3:1, 4:1, 5:1, and 6:1 (aqueous:organic) were evaluated.

**Figure 4:**
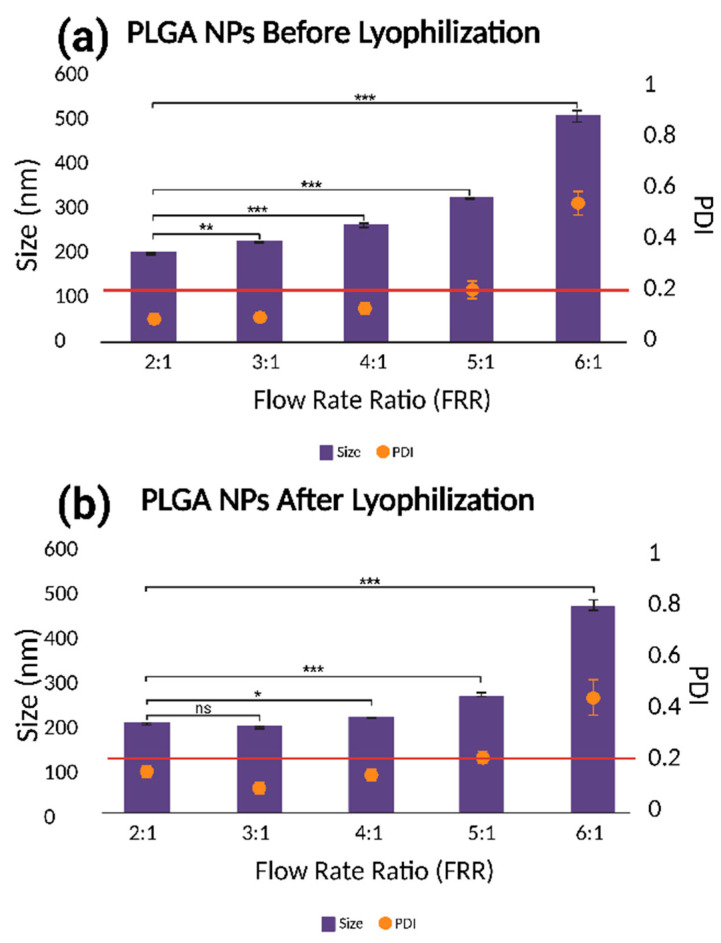
The effect of aqueous:organic FRR on the PLGA NP size and polydispersity index (PDI) obtained (**a**) before and (**b**) after lyophilization (ns = nonsignificant, * = *p* < 0.05, ** = *p* < 0.01, *** = *p* < 0.001).

As shown in Figure 4, the hydrodynamic size and PDI of PLGA NPs increase progressively with the flow rate ratio (FRR), both before (Figure 4a) and after (Figure 4b) lyophilization. This trend is particularly pronounced at an FRR of 6:1, where a sharp rise in both the size (>500 nm) and PDI (~0.8) is observed, indicating a poor size control and particle heterogeneity. Notably, the 3:1 FRR condition deviates from this trend after lyophilization, showing a slight reduction in the size compared to 2:1, along with a consistently low PDI (<0.2), suggesting improved particle stability.

The lyophilization process itself contributes to an increase in the particle size and PDI due to nanoparticle aggregation and heterogeneous redispersion upon rehydration [39,40]. Despite these effects, FRRs of 2:1 to 4:1 consistently produce nanoparticles with diameters below 250 nm and narrow size distributions, indicating good formulation robustness.

FRR, defined as the ratio of aqueous to organic flow rates, plays a crucial role in nanoprecipitation. As the FRR increases, the organic phase is diluted more rapidly, accelerating polymer precipitation and nucleation, which can initially favor monodisperse particle formation [41]. However, this relationship is modulated by the architecture of the microfluidic mixing channel. At lower FRRs, the herringbone channel designs enhance the mixing efficiency by increasing the interfacial area and shear forces, leading to smaller, more uniform nanoparticles. In contrast, at higher FRRs, the influence of the channel geometry diminishes, resulting in less controlled mixing and larger particles [42]. Overall, among the tested conditions, FRRs of 2:1 and 3:1 provide the optimal balance of the particle size, monodispersity, and stability following lyophilization, and were therefore selected for subsequent optimization studies.

**Figure 5:**
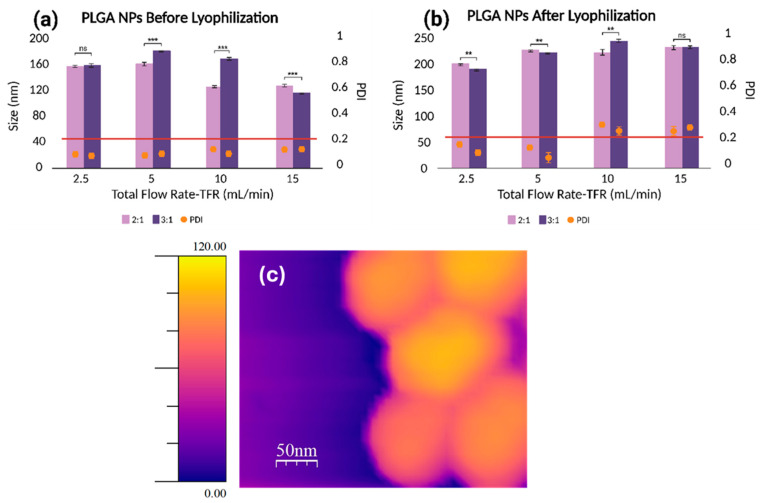
The effect of aqueous+organic TFRs on the PLGA NP size and polydispersity index (PDI) obtained (**a**) before and (**b**) after lyophilization at 2:1 (light purple) and 3:1 (dark purple) FRRs (ns = nonsignificant, ** = *p* < 0.01, *** = *p* < 0.001). (**c**) Atomic force microscopy height image of PLGA NPs produced at 3:1 FRR and 2.5 mL/min TFR.

The total flow rate (TFR), defined as the sum of the aqueous and organic flow rates, is a critical parameter influencing the PLGA nanoparticle size, size distribution, and production efficiency in microfluidic systems [43]. An increase in TFR elevates the liquid velocity within the microchannel, thereby shortening the mixing time and promoting rapid nucleation, which typically results in smaller, more uniform nanoparticles [44]. However, excessively high TFRs might lead to insufficient mixing and incomplete particle formation as the fluids exit the microchannel in a premature manner [45]. Therefore, optimizing TFR is essential for achieving desirable nanoparticle characteristics. To evaluate this effect, TFRs of 2.5, 5, 10, and 15 mL/min were tested while keeping the flow rate ratios (FRRs) fixed at 2:1 and 3:1. As shown in Figure 5a, increasing the TFR generally led to a decrease in the nanoparticle size, consistent with the literature [15,42]. Notably, size differences between FRRs of 2:1 and 3:1 became statistically significant at TFRs ≥ 5 mL/min while PDI values remained below 0.2 across all conditions. After lyophilization (Figure 5b), however, the trend diverged. Both the nanoparticle size and PDI increased with TFR at both FRRs. This suggests that while higher TFR improves mixing and particle formation prior to drying, the lyophilization and rehydration processes may destabilize these smaller particles, leading to aggregation and heterogeneity. The differences in the size between FRRs remained significant at lower TFRs (2.5 and 5 mL/min) but became statistically non-significant at 15 mL/min, indicating diminished control at higher flow conditions. The hydrodynamic size and PDI of PLGA nanoparticles after lyophilization are especially relevant for downstream applications such as drug delivery. Nanoparticles within the 20–200 nm range have been shown to preferentially accumulate in tumor tissue via the enhanced permeability and retention (EPR) effect, enabling improved therapeutic outcomes [46,47]. Although no strict PDI threshold is universally accepted, a PDI below 0.2 is commonly used as a benchmark for monodispersity in nanoparticle formulations [48].

Overall, the formulation prepared at FRR of 3:1 and TFR of 2.5 mL/min exhibited the smallest size and most uniform size distribution. It should be noted that we used DLS as the primary technique for rapid measurement of nanoparticle populations in their native dispersed state; however, this method has well-recognized limitations [49]. Because scattering intensity scales with the sixth power of particle size, DLS is inherently biased toward larger particles or aggregates, and intensity-weighted Z-averages may overestimate mean size. As emphasized by Filippov et al. [49], comparison of number- and volume-weighted distributions offers a more reliable assessment. In our study, the volume-weighted distributions aligned well with the intensity-averaged values, particularly for nanoparticles with PDI < 0.2, which we used as a quality criterion for acceptable formulations. Moreover, DLS assumes spherical geometry and reports an apparent hydrodynamic diameter, which can differ from the physical size determined by imaging techniques. The atomic force microscopy (AFM) imaging of the optimized formulation provided complementary information on morphology, confirming a spherical shape with uniform spacing and alignment (Figure 5c and Figure S1). These characteristics identify the formulation as the most favorable candidate for further optimization and potential therapeutic applications.

#### The Impact of Mixer Inlet Geometry on the Colloidal Properties of PLGA NPs

To evaluate the effect of mixer inlet geometry on the colloidal properties of PLGA NPs, we compared particles formulated using a three-inlet junction mixer with those produced using a Y-junction mixer. Both setups used the same formulation parameters (10 mg/mL of PLGA in ACN with 1% PVA) and identical flow conditions (FRR 3:1, TFR 2.5 mL/min). As shown in Table 4, both mixers produced NPs with similar sizes and PDI values before lyophilization. After lyophilization, the difference became more pronounced. While the three-inlet junction system showed only a modest increase in the particle size and maintained a low PDI, the Y-junction mixer resulted in a larger size increase (200.6 nm) and a borderline PDI value (0.240), approaching the upper limit for acceptable monodispersity.

These differences can be attributed to the influence of the inlet geometry on the mixing dynamics. To better understand these effects, we performed simulations for both mixers under the optimal operating conditions (flow rate ratio [FRR] = 3:1 and total flow rate [TFR] = 2.5 mL/min), while imposing atmospheric pressure condition at the outlet. The mesh characteristics used for both geometries are summarized in Table 5.

To estimate the mean diameter of PLGA nanoparticles from the outlet concentration obtained in our CFD simulations, it is necessary to combine the molar concentration with the particle number concentration under the assumption of monodisperse, spherical particles [50]. If $c_{m}\left(\frac{kg}{m^{3}}\right)$ is the mass of polymer per unit volume and $N\;\left(particles/m^{3}\right)$ is the total number of particles in that same volume, then each particle occupies an average volume;

(5) $$V_{p}=\frac{c_{m}}{N\rho_{p}}$$

where $\rho_{p}\;\left(\frac{kg}{m^{3}}\right)$ is the density of the solid polymer. Treating each particle as a perfect sphere, the volume can be expressed as;

(6) $$V_{p}=\frac{\pi }{6}d^{3}$$

Combining these two above expressions gives the following working formula [50]:

(7) $$d={\left(\frac{6}{\pi }\frac{c_{m}}{N\rho_{p}}\right)}^{1/3}$$

In applying Equation (7), we set a baseline particle number concentration of $N\approx 7\times 10^{17}\;\left(particles/m^{3}\right)$; the procedure used to infer this value and the accompanying sensitivity analysis are provided in Supplementary Information file. Table 6 presents the mean PLGA nanoparticle diameters obtained for both the three-inlet junction and Y-junction geometries, calculated from their respective inlet and outlet molar concentrations (using Equation (7)).

A mesh-independence study was conducted by refining the discretization to approximately 6 million elements for the three-inlet junction mixer and 20 million elements for the Y-junction system. Despite this substantial increase in the mesh resolution, the predicted outlet molar concentration—and hence the computed mean nanoparticle diameter remained essentially unchanged (the difference was less than 2%). These results confirm that the original mesh densities were sufficient, and the solution is mesh-independent.

**Figure 6:**
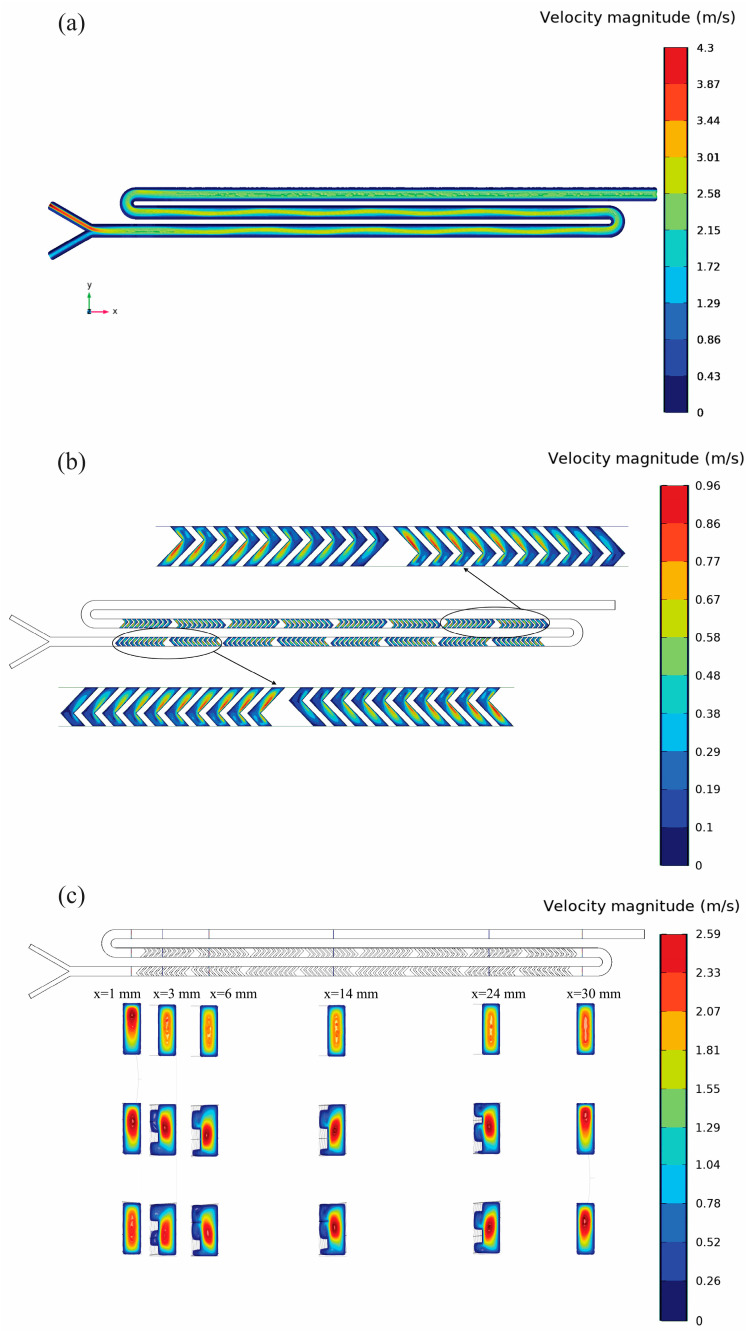
Velocity distribution within the Y junction mixer for FRR 3:1 and TFR 2.5 mL/min. (**a**) Velocity magnitude contours at the central plane (z = 0.1 mm), (**b**) Zoomed velocity contours at the lower plane (z = –0.05 mm) within the herringbone grooves, highlighting alternating zones of elevated and reduced velocity that generate secondary vortices to enhance local mixing, and (**c**) Cross-sectional velocity maps at six axial stations (x = 1, 3, 6, 14, 24, and 30 mm). Near the inlet (x = 1–6 mm) the cores show strong recirculating “cavity flows” adjacent to the herringbone tips—evidence of the vortical rolls that drive lateral convection. Farther downstream (x = 14–30 mm) these cavity vortices weaken and the contours become more symmetric, signaling flow re-stabilization once mixing has largely plateaued.

To further investigate these effects, the velocity distributions and PLGA concentration contours in both devices were obtained using CFD simulations. Figure 6 illustrates the velocity distributions inside the Y-junction mixing device at various planes and cross-sectional locations, describing the pivotal relationship between the channel geometry and the fluid flow as well as their impact on the nanoparticle formation.

In Figure 6a, velocity magnitude contours at the central plane (z = 0.1 mm) illustrate that velocities near the Y-junction inlet are initially high, indicating vigorous early-stage interactions between aqueous and organic phases. As the fluid moves downstream, the velocity magnitudes gradually decrease, transitioning towards laminar flow conditions (Re ≅ 200). This behavior represents that reduced mixing efficiency has occurred further downstream, which could lead to nanoparticle nucleation that is heterogeneous and size distributions that are more diverse at the outlet. Figure 6b depicts the velocity magnitude contours on the lower plane (z = −0.05 mm), particularly inside the embedded herringbone structures. At this location, alternating regions of high and low velocity clearly show local secondary flows along with vortical motion generated by the geometric disturbances. These local flow structures are the main reason for the large increase in mixing because they repeatedly fold and stretch layers of fluid. Although these local changes are positive contributions, the global mixing capacity is still confined by the Y-shaped geometry overall.

**Figure 7:**
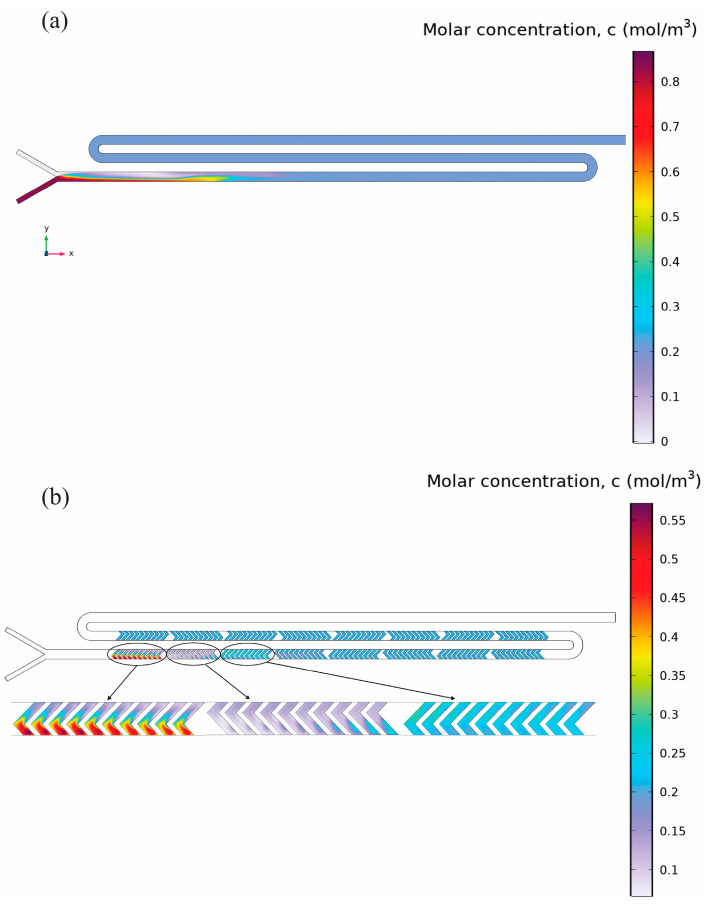
PLGA molar concentration distribution in the Y-junction mixer under FRR 3:1 and TFR 2.5 mL/min. (**a**) Central-plane (z = 0.1 mm) contours: the sharp red–blue interface at the inlet shows the initial unmixed streams, followed by gradual axial diffusion highlighted by the shifting color band, resulting in an outlet average of ~0.21 mol/m^3^. and (**b**) Detailed concentration contours at the lower plane (z = –0.05 mm) within the herringbone structures. Upstream herringbones (left) generate strong alternating high/low streaks due to the secondary vortices. Mid-channel herringbones (center) show blended color gradients (green/yellow), indicating progressive mixing. The furthest downstream herringbone grooves continue to display subtle concentration gradients, indicating that complete homogenization remains unachieved despite multiple cycles of interface deformation.

Furthermore, velocity profiles at six geometrically different (yz) sections throughout the channel (x = 1, 3, 6, 14, 24, and 30 mm) shown in Figure 6c confirms this pattern. Primary cross-sections (x = 1 and 3 mm) display typical central velocity peaks that are in agreement with the initial mixing conditions. Downstream positions (x = 6 and 14 mm) outline asymmetric flow patterns that emerge from the increased secondary vortices generated by the herringbones. At the last part of the channel (x = 24 and 30 mm), the flow tends to be more symmetric and stabilizes substantially, although residual mixing limitations remain evident. Collectively, Figure 6 underlines the complex interaction between geometry-induced flow patterns and mixing efficiency and shows that localized geometric enhancements such as herringbones alone cannot fully offset inherent mixing limitations of the Y-shaped junction, which ultimately impacts the nanoparticle size uniformity and stability. Figure 7 illustrates PLGA molar concentration distributions, explicitly links the upstream flow dynamics shown in Figure 6 to the downstream mixing behavior and concentration homogenization and nanoparticle formation results.

Figure 7a shows the concentration contours at z = 0.1 mm. The figure clearly shows an initial sharp concentration gradient at the inlet junction due to limited mixing of organic and aqueous streams. Further downstream the channel, the gradients become less pronounced, but even the outlet is not completely homogenized in terms of concentration. The average concentration of PLGA at the outlet, which was measured experimentally, is 0.21 mol/m^3^. Figure 7b specifically displays concentration distributions within herringbone structures at the lower plane (z = −0.05 mm). Here, distinct alternating high- and low-concentration patterns together with vortical flow regions depicted previously. These local mixing enhancements progressively homogenize the concentration but are insufficient to achieve uniformity along the channel’s entire length. When considering Figure 6 and Figure 7a,b are closely associated with the dependency of concentration distributions on flow patterns. While there are significant local improvements in the mixing efficiency, global constraints caused by the Y-junction still remain, leading to wider nanoparticle size distributions and possibly larger particles, which highlights the need for an optimal geometry.

**Figure 8:**
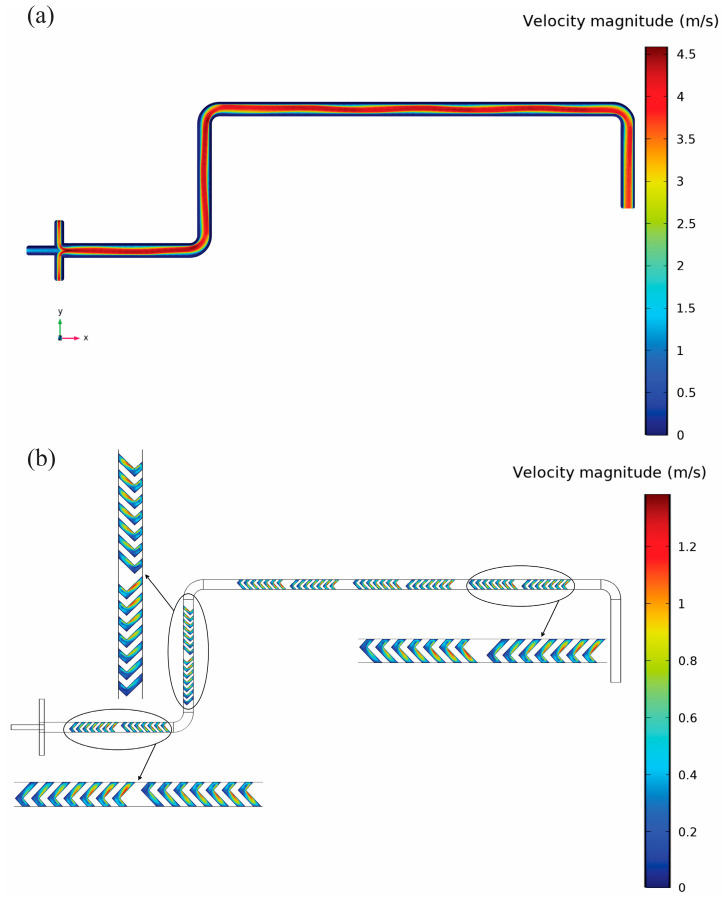
Velocity distribution within the three-inlet junction mixing device under FRR 3:1 and TFR 2.5 mL/min. (**a**) Contours at the mid-height plane (z = 0.1 mm) show nearly uniform, high-velocity flow from the symmetrical junction through the entire serpentine channel. The red core indicates the fastest streamlines, while the thin blue boundary layer at all walls signifies fully developed laminar flow. (**b**) Detailed lower-plane (z = –0.05 mm) contours in the herringbone grooves show alternating high and low velocity bands that drive secondary vortical flows and boost local mixing.

Figure 8 shows the velocity distributions within the three-inlet junction mixer as well as the symmetrical geometry and embedded herringbone structures.

**Figure 9:**
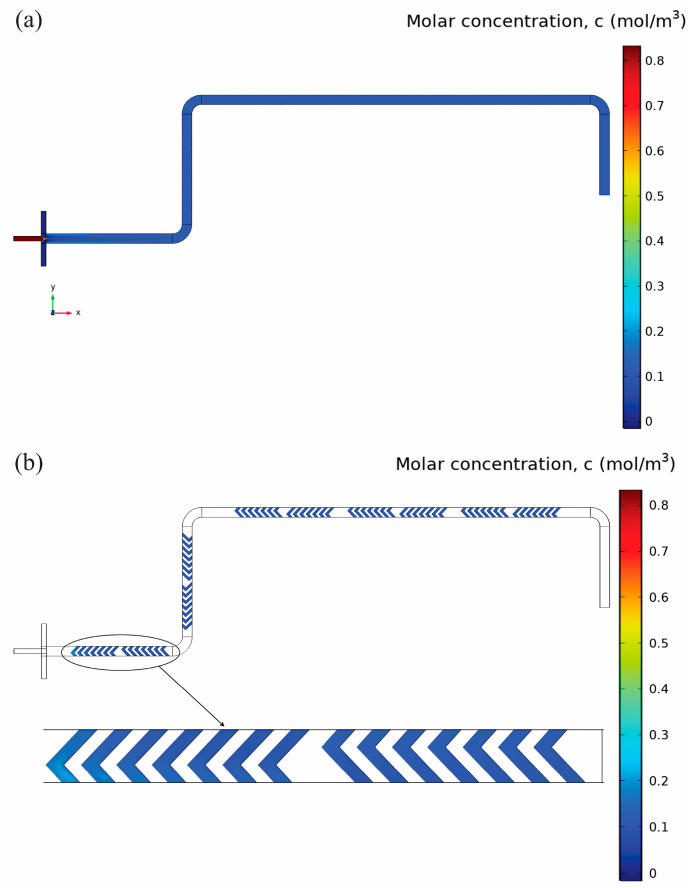
Contours of PLGA concentration in the three-inlet junction mixer at FRR 3:1 and TFR 2.5 mL/min, (**a**) Central-plane contours (z = 0.1 mm) show near-complete homogenization of PLGA immediately after the symmetric junction: the red core from the organic inlet is rapidly diffused into a uniform blue field, indicating an outlet average of ~0.12 mol/m^3^. (**b**) Detailed view at the bottom plane (z = –0.05 mm) within the first herringbone elements. The subtle, nearly uniform color gradient here confirms that secondary vortices generated by the herringbones have fully mixed the interface by this stage.

Figure 8a depicts the velocity contours at the center plane (z = 0.1 mm) that reveal the superior mixing performance just beyond the three-inlet junction. The symmetrical design ensures that the flow convergence is balanced so that high and uniform velocities are sustained along the whole channel which is very different from the patterns of the Y-junction. A local velocity distribution in the herringbone structures at the lower plane (z = −0.05 mm) is also displayed in Figure 8b. The alternating velocity regions clearly indicate the great induction of secondary flows and vortices. As a result, the local mixing efficiency is greatly improved. In short, Figure 8a,b clearly illustrate how the symmetrical inlet design together with the geometric patterns positively manipulate flow dynamics, thus not only more efficient and uniform mixing but also controlled nanoprecipitation becomes possible. Figure 9 demonstrates PLGA concentration distributions within the three-inlet junction mixer, explicitly linking optimized flow patterns from Figure 8 to concentration homogenization results.

At the central plane (z = 0.1 mm) in Figure 9a, the concentration contours reveal rapid, uniform mixing along with high and balanced velocities. The concentration gradients are small all along the channel length, and the concentration at the outlet is about 0.12 mol/m^3^ on average, which is about 42% less than the Y-junction mixer. The concentration contours in Figure 9b at the lower plane (z = −0.05 mm) within the herringbone structures are in perfect agreement with this result. The uniform and low concentration gradients that do not change in time serve as evidence for the better local mixing performance, which is due to the effect of the vortices caused by the herringbone structures. Figure 9a,b thus clearly prove the direct relationship among the optimized geometry, efficient velocity distributions, and resulting superior concentration homogeneity, which explains the enhanced nanoparticle formation performance in the three-inlet junction mixer, namely smaller particle sizes and narrower distributions.

To summarize, the three-inlet junction provides more symmetrical and efficient mixing, enhancing interfacial contact between the aqueous and organic phases, which promotes rapid and uniform nanoprecipitation, resulting in smaller and more stable nanoparticles. In contrast, the Y-junction geometry might result in less efficient mixing and uneven flow convergence, leading to broader particle size distributions and reduced structural stability after lyophilization. While our results highlight a significant difference between the Y-junction and three-inlet junction systems, we note that this comparison also involves secondary variations in channel length and spacing. Thus, the performance differences cannot be attributed solely to the number of inlets but to the overall inlet design and associated flow geometry. Future studies should control these parameters more rigorously to isolate the effect of inlet number. These findings highlight the critical role of microfluidic design in achieving clinically desirable nanoparticle features.

## 4. Conclusions

This study employs systematic experimentation and advanced computational modeling to optimize PLGA nanoparticle formulation through both batch and microfluidic nanoprecipitation methods. Design of Experiments (DoE) guided the identification of key formulation parameters in the batch process, while microfluidic preparation enabled precise control over mixing conditions, leading to smaller and more uniform nanoparticles. A detailed comparison between two microfluidic mixer geometries, namely Y-junction and three-inlet junction mixers, revealed that the inlet design significantly affects colloidal outcomes, particularly following lyophilization. Computational fluid dynamics (CFD) simulations offered critical insights into these differences, proving how symmetrical flow fields and enhanced mixing in the three-inlet system led to superior concentration homogenization and controlled nanoparticle formation. Due to the agreement of simulated outlet concentrations with experimentally ones, the computational model validated its predictive capacity.

Overall, our study highlights the importance of combining experimental optimization with fluid dynamics simulations to offer valuable guidelines for microfluidic device design and nanoparticle engineering. The findings establish a scalable and reproducible framework for producing clinically relevant PLGA nanoparticles using both batch and microfluidic nanoprecipitation approaches.

## Figures and Tables

**Table 1:** Summary of formulation parameters and resulting particle characteristics for three sequential DoE iterations.

Iteration		V_ACN_ [μL]	V_PVA_ [mL]	C_PLGA_ [mg]	C_PVA_ [%]	Size [nm]	Std Dev	PDI	Std Dev
1	*F1.1*	500	1	5	2.5	136	±1.0	0.07	±0.01
	*F1.2*	500	1	15	1.5	198	±1.0	0.10	±0.02
	*F1.3*	100	3	5	2.5	173	±1.0	0.07	±0.01
	*F1.4*	100	3	15	1.5	263	±5.0	0.32	±0.01
	*F1.5*	500	3	5	1.5	132	±1.0	0.05	±0.02
	*F1.6*	100	1	15	2.5	281	±3.0	0.29	±0.01
	*F1.7*	100	1	5	1.5	173	±1.0	0.08	±0.04
	*F1.8*	500	3	15	2.5	200	±1.0	0.10	±0.02
2	*F2.1*	100	3	15	1.5	270	±9.0	0.27	±0.04
	*F2.2*	100	1	5	1.5	175	±1.0	0.08	±0.01
	*F2.3*	100	3	5	2.5	194	±1.0	0.07	±0.04
	*F2.4*	500	1	15	1.5	199	±2.0	0.09	±0.01
	*F2.5*	500	3	15	2.5	200	±1.0	0.07	±0.01
	*F2.6*	100	1	15	2.5	255	±2.0	0.18	±0.02
	*F2.7*	500	3	5	1.5	134	±1.0	0.05	±0.02
	*F2.8*	500	1	5	2.5	140	±1.0	0.06	±0.02
3	*F3.1*	100	3	12	1.5	1483	±820	0.84	±0.14
	*F3.2*	100	1	12	2.5	4793	±834	0.91	±0.08
	*F3.3*	500	3	12	2.5	208	±1.0	0.24	±0.02
	*F3.4*	500	3	5	1.5	124	±1.0	0.15	±0.01
	*F3.5*	500	1	12	1.5	195	±2.0	0.06	±0.01
	*F3.6*	500	1	5	2.5	156	±1.0	0.06	±0.03
	*F3.7*	100	3	5	2.5	253	±1.0	0.29	±0.02
	*F3.8*	100	1	5	1.5	273	±3.0	0.38	±0.02

**Table 2:** Regression model summary for particle size: coefficients of determination (R^2^) and *p*-values of key formulation factors across three DoE iterations.

Iteration	Size R^2^	*p*_PLGA/ACN_	*p*_%PVA_	*p*_Aq/Org_
1	0.888	* 0.007	0.725	0.379
2	0.842	* 0.020	0.237	0.756
3	0.908	* 0.004	0.139	* 0.039

* *p* < 0.05.

**Table 3:** Regression model summary for PDI: coefficients of determination (R^2^) and P-values of key formulation factors across three DoE iterations.

Iteration	PDI R^2^	*p*_PLGA/ACN_	*p*_%PVA_	*p*_Aq/Org_
1	0.172	0.698	0.468	0.754
2	0.899	* 0.009	0.108	0.847
3	0.967	* 0.001	0.273	0.591

* *p* < 0.05.

**Table 4:** Size and polydispersity index (PDI) of PLGA NPs prepared at FRR 3:1, TFR 2.5 mL/min using two mixers featuring a Y-junction and a three-inlet junction.

Inlet Geometry	Before Lyophilization		After Lyophilization	
	Mean Diameter (nm)	PDI	Mean Diameter (nm)	PDI
Y-junction	158.1 ± 0.6	0.101	200.6 ± 2.2	0.240
Three-inlet junction	157.5 ± 2.6	0.084	173.1 ± 1.5	0.141

**Table 5:** Mesh statistics for the computational domain of the two microfluidic mixers used for PLGA nanoparticle preparation.

Inlet Geometry	Number of Elements	Minimum Element Quality	Average Element Quality	Element Volume Ratio
Three-inlet junction	3,414,891	0.06555	0.676	6.21 × 10^−4^
Y-junction	11,330,936	0.06417	0.6803	5.21 × 10^−6^

**Table 6:** Inlet/outlet PLGA concentrations and mean nanoparticle diameters for the two microfluidic mixers.

Inlet Geometry	Molar Concentration of PLGA at Inlet (mol/m^3^)	Molar Concentration of PLGA at Outlet (mol/m^3^)	Mean Diameter (nm)
Three-inlet junction	0.83	0.12	171
Y-junction	0.83	0.21	206

## Data Availability

The original contributions presented in this study are included in the article.

## Supplementary Materials

The following supporting information can be downloaded at: https://www.mdpi.com/article/10.3390/mi16090972/s1, Figure S1: AFM height image of PLGA NPs produced at 3:1 FRR and 2.5 mL/min TFR. Scan size: 2 µm × 2 µm; supplementary information on Mapping Outlet Concentration to Mean Particle Diameter: Derivation, Calibration, and Sensitivity.
