# Supplementary material for: Optimization of PLGA Nanoparticle Formulation via Microfluidic and Batch Nanoprecipitation Techniques
Source: Micromachines (Basel). 2025 Aug 24;16(9):972. doi: 10.3390/mi16090972 (PMC12471678; doi:10.3390/mi16090972)
Supplement: Supplementary file 1 [file micromachines-16-00972-s001.zip › micromachines-3798903-supplementary.pdf]

Supplementary Information File
Optimization of PLGA Nanoparticle Formulation via Microfluidic and Batch Nanoprecipitation Techniques

Gül Kozalak, Salar Heyat Davoudian, Evangelos Natsaridis, Nubia Gogniat, Ali Koşar, Oya Tagit

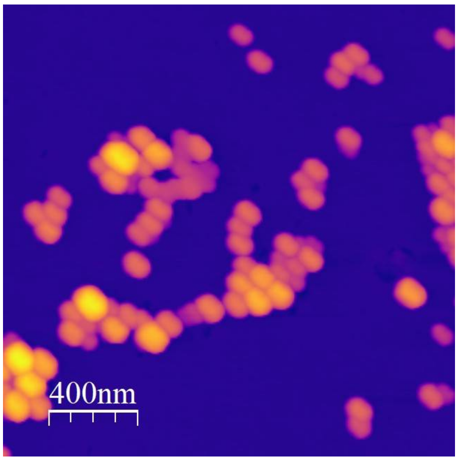

Figure S1. AFM height image of PLGA NPs produced at 3:1 FRR and 2.5 mL/min TFR. Scan size: 2 µm x 2 µm.

Mapping Outlet Concentration to Mean Particle Diameter: Derivation, Calibration, and Sensitivity

1. Governing relations and units

To relate CFD-predicted outlet concentration to the mean particle diameter d of PLGA nanoparticles, we adopt the standard assumptions of spherical and monodisperse particles and close the mass balance at the outlet control volume. Let

- $c_m \left(\frac{kg}{m^3}\right)$ be the mass concentration of polymer per unit volume,
- $N\ (particles/m^3)$ the total number of particles in that same volume,
- $\rho_p \left(\frac{kg}{m^3}\right)$ the density of the solid polymer.

The average particle volume follows from mass conservation:

$$V_p = \frac{c_m}{N\rho_p} \tag{1}$$

Assuming particles as perfect spheres ($V_p = \frac{\pi}{6}d^3$) gives the working relation:

$$d = \left(\frac{6}{\pi}\frac{c_m}{N\rho_p}\right)^{1/3} \tag{2}$$

When CFD provides molar outlet concentration $c_{out}\ (mol \cdot m^{-3})$, we convert to mass units via

$$c_m = M_{eff}c_{out}f \tag{3}$$

where $M_{eff}\ (kg \cdot mol^{-1})$ is an effective molar mass consistent with the PLGA feed specification and $f \in (0,1]$ is the precipitated fraction (here taken as $f = 1$ unless stated otherwise). Results scale as $d \propto {M_{eff}}^{1/3}$ and $d \propto f^{1/3}$.

2. Data inputs (Table 6)

We use the experimentally reported mean diameters together with CFD outlet molar concentrations:

Inlet geometry	Molar concentration of PLGA at inlet (mol/m³)	Molar concentration of PLGA at outlet (mol/m³)	Mean diameter (nm)
Three-inlet junction	0.83	0.12	171
Y-junction	0.83	0.21	206

3. Baseline calibration of number concentration

Inverting Eq. (2) gives

$$N = \frac{6}{\pi}\frac{c_m}{\rho_p d^3} \tag{4}$$

Using the Table 6 diameters and c_m from Eq. (3) (with $\rho_p$taken from PLGA literature and a consistent M_{eff}, we obtain order-of-magnitude baseline values $N \approx 7 \times 10^{17}\ particles/m^3$ for both the three-inlet and Y-junction mixers.

4. Sensitivity of diameter to number concentration *N*

From Eq. (2),

$$d \propto N^{-1/3} \tag{5}$$

Thus, multiplying N by a factor r scales the diameter by $r^{-1/3}$. We quantify decade-scale uncertainty by sweeping N over {0.1, 0.25, 0.5, 1, 2, 4, 10}×N while holding c_m and ρ_p fixed.

Tamara (three-inlet) mixer, Molar concentration of PLGA at outlet=0.12 mol/m³, and Mean diameter=171nm

N setting	Mean Diameter (nm)	Δ vs Baseline (%)
$0.1N$	368.40	-115
$0.25N$	271.44	-58
$0.5N$	215.44	-26
N	171	0
$2N$	135.72	20
$4N$	107.72	37
$10N$	79.37	53

Y-junction mixer, Molar concentration of PLGA at outlet=0.21 mol/m³, and Mean diameter=206nm

N setting	Mean Diameter (nm)	Δ vs Baseline (%)
$0.1N$	443.81	-115
$0.25N$	327	-58
$0.5N$	259.54	-26
N	206	0
$2N$	163.5	20
$4N$	129.77	37
$10N$	95.61	53

This analysis shows the expected cubic-root law $d \propto N^{-1/3}$: halving/doubling N changes d by $\pm\approx 26\%/20\%$, quartering/quadrupling by $\pm\approx 59\%/37\%$, and a ten-fold decrease/increase by $\approx +115\%/-54\%$—effects that adjust absolute diameters but leave the relative ordering intact. Crucially, the inter-mixer ratio which matches the observed $206/171\ \approx\ 1.205$, indicating that the smaller size in the three-inlet mixer is a direct consequence of its lower outlet concentration (better mixing/anti-solvent dilution) rather than numerical artifacts. Thus, while Eq. 7 in the main text should not be treated as a point-predictor without an independent N measurement, the sensitivity results demonstrate that our central conclusion—the three-inlet geometry yields smaller, more uniform particles than the Y-junction—is robust to plausible decade-scale uncertainty in N.
